# Roasting-Induced Changes in Bound Phenolics and Their Contribution to Antioxidant Activity in Barley Malts

**DOI:** 10.3390/antiox15070815

**Published:** 2026-06-28

**Authors:** Guo-Dong Zhuang, Wen-Ting Gu, Hua-Ze Wu, Ao Li, Dan Tang, Yong-Sheng Chen

**Affiliations:** 1 Department of Food Science and Engineering, Jinan University, Guangzhou 510632, China; guodong0310@126.com; 2 Key Laboratory of Digital Quality Evaluation of Chinese Materia Medica of State Administration of TCM, Guangdong Pharmaceutical University, Guangzhou 510006, China

**Keywords:** malt, bound phenolics, thermal processing, UPLC-Q-Orbitrap HRMS/MS, DPPH-UPLC, AAPH-UPLC, antioxidant activity

## Abstract

Background/Objectives: This study investigated the effects of different roasting levels on bound phenolic composition and antioxidant activity in barley malts. Methods: Bound phenolics from raw, fried, and dark malts were characterized using UPLC-Q-Orbitrap HRMS/MS combined with multivariate analysis. Potential antioxidant-active compounds were screened by DPPH/AAPH-incubating UPLC-DAD and further evaluated by in vitro antioxidant assays. Results: A total of 44 bound phenolic compounds were tentatively characterized. Distinct differences in bound phenolic profiles were observed among the three malts, with raw malt exhibiting relatively higher levels of bound phenolics. PCA analysis revealed compositional differences among samples. Activity-guided screening identified epicatechin gallate, trans-ferulic acid, and cis-ferulic acid as compounds exhibiting pronounced radical-scavenging activity, which was further supported by in vitro assays. Conclusions: Different roasting levels were associated with changes in bound phenolic composition and antioxidant properties of barley malts, providing insights into the relationship between thermal processing and the functional quality of malt-based products.

## 1. Introduction

Barley, among the world’s most ancient cereal crops, holds the fourth position in global grain output [1,2]. This versatile grain is chiefly employed in food processing, malt generation, beverage fermentation, and livestock feed. With a cultivation history exceeding three millennia in China, barley serves as a vital economic crop. In Chinese practices, barley kernels are sprouted and dehydrated to yield unprocessed malt, a substance long valued for its therapeutic and dietary benefits [3]. The composition of raw malt includes phenolic acids, flavonoid derivatives, enzymatic proteins, tannic substances, proanthocyanidin polymers, and aminophenolic constituents, the concentrations of which fluctuate depending on heat treatment conditions [4]. Roasting promotes the liberation of phenolic constituents, driven by enzymatic action and glycosylation processes. Given rising consumer demand for functional foods, barley malt has gained significant traction in the nutraceutical sector owing to its rich bioactive profile.

Recent decade has witnessed growing scientific interest in cereal processing byproducts as valuable reservoirs of phytochemicals. These agricultural residues contain abundant phenolic metabolites, a class of biologically active secondary products crucial for regulating oxidative balance in living systems [5,6]. As key secondary metabolites in plants, phenolic constituents significantly influence food quality parameters, primarily through their ability to scavenge reactive oxygen species and chelate prooxidant metals [6]. These compounds effectively scavenge reactive oxygen species and terminate oxidative chain reactions, making them indispensable functional components [7,8,9]. In plant metabolism, phenolic compounds serve dual roles as both antioxidant agents and growth-regulating phytochemicals [10]. These compounds are conventionally classified based on their extractability into two forms: soluble phenolics, which can be isolated through polar solvent extraction, and bound phenolics that require hydrolytic cleavage via alkaline, acidic, or enzymatic treatments for liberation [11].

Bound phenolics are ubiquitously distributed across plant-derived food matrices, particularly in vegetables, fruits, cereals, legumes, and nuts [12]. Although research has traditionally focused on soluble phenolics because of their higher bioaccessibility, increasing attention has been paid to bound phenolics in recent years. Previous studies have demonstrated that bound phenolics can make substantial contributions to antioxidant activity; however, their relative importance compared with soluble phenolics varies depending on the plant matrix, extraction procedures, and antioxidant assays employed [13,14,15,16]. In our previous study, bound phenolic fractions from thermally processed barley malt products exhibited higher phenolic contents and stronger antioxidant activity than the corresponding soluble fractions under the specific experimental conditions investigated [4]. These findings prompted the present study focusing on the compositional characteristics and antioxidant properties of bound phenolics in barley malts.

In our previous study, we found that bound phenolics in three malts exhibited high antioxidative activities both in vitro and in cellular assays [4]. The DPPH-HPLC method, leveraging ligand-enzyme interaction principles, provides a rapid, efficient, and high-throughput approach for identifying antioxidant constituents in complex natural matrices [17]. This technique is particularly suited for phenolic compound screening due to their characteristic DPPH radical-scavenging properties. Furthermore, we pioneered an innovative AAPH-HPLC method for direct antioxidant evaluation in natural extracts [18]. The assay utilizes temperature-controlled (37 °C) decomposition of AAPH in phosphate buffer (pH 7.4) to produce peroxyl radicals, simulating biological oxidation conditions. Antioxidant activity is indicated by the reduction or elimination of the chromatographic peak areas, with subsequent structural elucidation achieved through high-resolution tandem mass spectrometry [19].

This investigation employed UPLC-Q-Orbitrap HRMS/MS to characterize thermal degradation kinetics of bound phenolic compounds in untreated raw malt, and to swiftly screen and identify antioxidant components in bound phenolics across three types of barley malt through DPPH-UPLC and AAPH-UPLC. This study provides new insights into the composition of bound phenolics in barley malt, offering a scientific basis for the development of nutraceuticals and functional foods enriched with antioxidant components.

## 2. Materials and Methods

### 2.1. Reagents

LC/MS-grade acetonitrile and formic acid were obtained from Fisher Scientific (Fair Lawn, NJ, USA). Reference standards—including trans-ferulic acid (≥98%), cis-ferulic acid (≥97%), *p*-coumaric acid (≥99%), sinapic acid (≥98%), vanillic acid (≥95%), homovanillic acid (≥97%), caffeic acid (≥98%), and catechin (≥95%) were purchased from Chroma-Biotech (Chengdu, China). All compounds were stored at −20 °C prior to analysis.

The study utilized three malt varieties, Raw malt (batch no.: 210301; Anhui origin), Fried malt (batch no.: 210201; Anhui), and Dark malt (batch no.: 200901; Anhui), procured from Guangzhou Zhixin Pharmaceutical Co., Ltd. (Guangzhou, China). Both Raw malt, Fried malt and Dark malt were sourced from the same manufacturer. For the production: Fried malt were Heated at 160–170 °C until the surface turned light brown-yellow. Dark malt were Heated at 190–200 °C until the surface turned dark brown.

### 2.2. Extraction of Bound Phenolic Compounds

The free and bound phenolic fractions were isolated following an adapted protocol based on a previously reported methodology [4,20]. An accurately measured portion (5.00 g) of the powdered sample was homogenized with 100 mL of an 80% (*v*/*v*) methanol-water solution, followed by ultrasonication (25 °C, 30 min). After centrifugation (2500× *g*, 10 min), which the liquid phase containing soluble phenolic constituents was removed. This extraction procedure was performed in triplicate to ensure complete elimination of free phenolics.

The residual solid was subjected to alkaline hydrolysis using 100 mL of 2 M NaOH, with constant shaking under nitrogen atmosphere at ambient temperature (25 °C) for 4 h to release bound phenolic compounds. Hydrolysis was performed under a nitrogen atmosphere to minimize oxidative degradation of phenolic compounds during alkaline treatment. Following hydrolysis, the pH of the reaction mixture was carefully neutralized to 2.0 by adding 6 M hydrochloric acid. The acidified solution then underwent multiple sequential extractions (n = 6) with ethyl acetate as the organic solvent. The ethyl acetate fractions were evaporated to dryness under reduced pressure at 45 °C, then redissolved in 5 mL of methanol. All prepared samples were maintained at −80 °C until subsequent analytical procedures.

Six independent replicates were prepared for each experimental sample. Prior to chromatographic analysis, the supernatants were subjected to filtration through 0.22 μm pore-size membranes and subsequently transferred to sterile centrifuge tubes. And then diluted to 0.5 mg/mL for analysis using UPLC and UPLC-Q-Orbitrap HRMS/MS. For quality assurance purposes, a composite quality control (QC) sample was generated by pooling equal aliquots (20 μL) from all bound phenolic fractions. This QC sample served as a system suitability check and was systematically analyzed after every third experimental injection throughout the analytical sequence. This approach ensured consistent instrument performance and data reliability during the extended analysis period [21,22].

### 2.3. Antioxidant Screening by DPPH/AAPH-Incubating UPLC-DAD

DPPH-Incubating UPLC-DAD was used to analyze the anti-oxidant compounds in the bound phenolics according to a previously published method with several optimizations [23]. Briefly, 1.0 mL of the bound phenolics were mixed with 1.0 mL of DPPH-methanol solution (5 mg/mL) and incubated in the dark for 30 min. Following incubation, the mixtures were membrane-filtered (0.22 μm) and the resulting filtrates were immediately subjected to UPLC analysis. As experimental controls, methanol-treated samples were processed identically and analyzed concurrently for direct comparison.

The antioxidant capacity of bound phenolic fractions against peroxyl radicals was evaluated using an AAPH-Incubating UPLC-DAD system [18]. For assay preparation, AAPH was dissolved in PBS (pH 7.4) at a concentration of 100 mg/mL (1.00 g in 10 mL). The reaction mixture, consisting of 1 mL bound phenolics and 2 mL AAPH solution (100 mg/mL), was vortex-mixed and incubated at 37 °C. Post-incubation, samples were filtered through 0.22 μm membranes prior to immediate UPLC analysis. As experimental controls, PBS-treated samples were processed identically and analyzed concurrently for direct comparison.

### 2.4. Measurement of the Antioxidant Activity by DPPH Radical Scavenging Assay

The radical scavenging activity was assessed through a modified DPPH assay following established methodology [24]. Test samples at varying concentrations were combined with 0.2 mM methanolic DPPH solution (1:1, *v*/*v*) and incubated under dark conditions at ambient temperature (25 °C) for 30 min. Methanol served as the blank control in all experiments. Reaction mixture absorbance measurements were obtained at 517 nm using a spectrophotometer. The components were identified as active components through DPPH-Incubating UPLC-DAD experiments, which screened the bound phenolics from the three types of malts.

### 2.5. Measurement of the Antioxidant Activity by ORAC Assay

The oxygen radical antioxidant capacity was evaluated using an optimized protocol adapted from prior methodology [24]. Experimental samples or Trolox calibration standards (50 μL aliquots) were combined with 100 μL of fluorescein working solution (0.96 μM final concentration) and equilibrated at 37 °C. Following a 20 min incubation period, 90 μL of freshly prepared AAPH radical initiator (26.55 mM) was introduced to both sample and standard wells. The fluorescence intensity change was recorded immediately at 485 nm excitation/535 nm emission for 35 cycles per 4.5 min using a Fluoroskan Ascent fluorescence spectrophotometer. The ORAC values were expressed as micromoles of Trolox equivalent per gram DW. The components were identified as active components through AAPH-Incubating UPLC-DAD experiments, which screened the bound phenolics from the three types of malts.

### 2.6. UPLC-Q-Orbitrap HRMS/MS Analysis

Separation was carried out using a Dionex Ultimate 3000 ultra-performance liquid chromatography system (Thermo Fisher Scientific, Waltham, MA, USA) integrated with an autosampler and a high-resolution Q-Exactive Orbitrap tandem mass spectrometer. The analytical separation was achieved on a BEH C18 reversed-phase column (100 mm × 4.6 mm, 1.8 μm) maintained at a constant temperature of 35 °C. The mobile phase was composed of: (A) 0.1% formic acid in water, and (B) methanol with a flow rate of 0.3 mL/min. The elution parameters were: 0–2 min, 5–5% B; 2–10 min, 5–15% B; 10–25 min, 35–50% B; 25–32 min, 50–50% B; followed by 5 min of re-equilibration.

Mass spectrometric analysis was conducted using a heated electrospray ionization source operating in positive or negative ion mode. The instrument parameters were optimized as follows: spray voltage at 3.5 kV, capillary temperature maintained at 350 °C, and auxiliary gas heater set to 100 °C. Gas flows were adjusted to 10 arbitrary units for the auxiliary gas and 45 arb for the sheath gas. Mass spectra were acquired across the range of *m*/*z* 100–1000.

### 2.7. Statistical Analysis

All experimental measurements were performed with three biological replicates. All UHPLC-MS/MS-derived raw data were analyzed using Compound Discoverer 3.0 software (Thermo Fisher, Waltham, MA, USA). Compound identification and relative quantification were achieved by aligning detected peaks with retention times, precise mass values, molecular formulas, MS/MS spectral interpretations, relevant literature, and entries in public repositories such as Metlin, HMDB, and mzCloud. A subset of the detected phenolic compounds was further verified by comparison with authentic standards [25]. And the resulting datasets were subjected to multivariate statistical analysis using SIMCA-P 14.1 (Umetrics AB, Umeå, Sweden), incorporating both unsupervised and supervised approaches. These methods were employed to track changes in bound phenolic compounds across various roasting temperatures of barley malts. PCA assessed the inherent variations in the data matrix and identified differences among samples of processed bound phenolics. OPLS-DA was utilized to classify samples based on Y variables alone. Critical compounds affecting bound phenolics during different roasting temperatures were identified using OPLS-DA, combined with a threshold for variable importance in projection (VIP > 1.0) and significance (*p* < 0.05) [26,27].

## 3. Results and Discussion

### 3.1. Global Chemical Profiling of Three Bound Phenolics Compounds Extract by UPLC-Q-Orbitrap HRMS/MS Analysis

**Figure 1:**
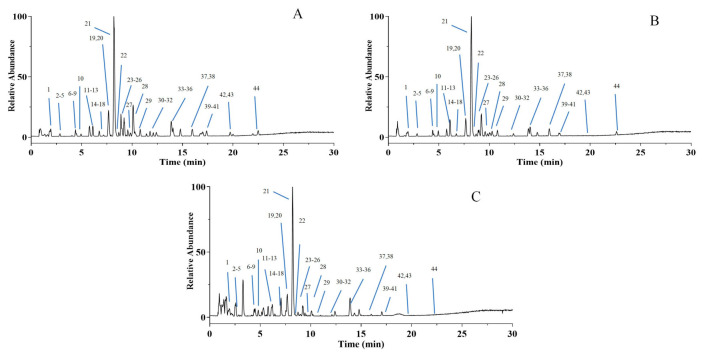
Total ion chromatograms (TIC) of the bound phenolics extracted by alkaline hydrolysis in raw malt (**A**), fried malt (**B**) and black malt (**C**) samples in negative modes.

**Figure 2:**
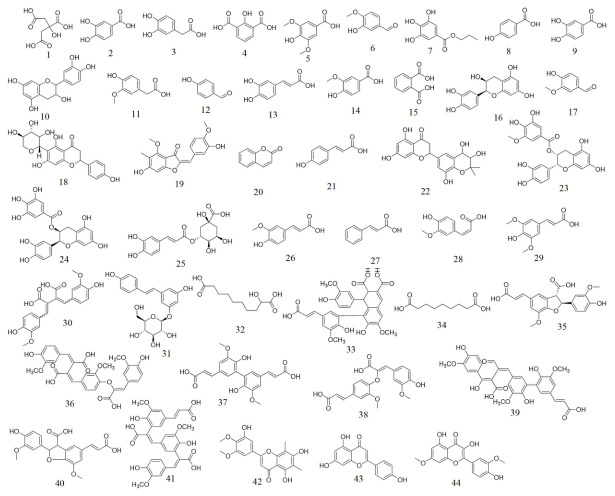
The structural formula of the bound phenolics extracted by alkaline hydrolysis in three malt.

This investigation employed UPLC-Q-Orbitrap HRMS/MS for the comprehensive characterization of bound phenolics released through alkaline hydrolysis from three barley malt varieties. Analyses were performed in negative ion mode to obtain molecular and fragmentation information [15]. Analytical measurements were performed in negative ion mode to examine molecular and fragment ion profiles. By comparing retention times, accurate mass measurements, MS/MS fragmentation patterns, authentic standards, and published literature, a total of 44 representative phenolic compounds were tentatively characterized [13,14,28]. According to the Metabolomics Standards Initiative (MSI) guidelines, compounds verified using authentic standards were assigned as level 1 identifications, whereas the remaining compounds were considered putatively annotated metabolites (level 2). The complete annotation results are summarized in Table 1, and the corresponding total ion chromatograms are shown in Figure 1. The structural formula of the bound phenolics extracted by alkaline hydrolysis in three malt are shown in Figure 2.

Three major constituents in the bound phenolics were characterized based on their retention times and mass spectrometric data (Table 1). Among them, compound 20 (tR = 8.21 min) exhibited a deprotonated molecular ion [M-H]^−^ at m/z 163.0392. Its MS/MS spectrum displayed characteristic fragment ions at *m*/*z* 119.0490 ([M-H-CO_2_]^−^), *m*/*z* 107.0494 ([M-H-2CO]^−^), and *m*/*z* 93.0333 ([M-H-2CO-CH_2_]^−^), consistent with *p*-coumaric acid. Compounds 25 (tR = 9.20 min) and 27 (tR = 10.10 min) both showed a deprotonated molecular ion [M-H]^−^ at *m*/*z* 193.0500. Their MS/MS spectra featured key fragments at *m*/*z* 134.0366 ([M-H-CO_2_-CH_3_]^−^) and *m*/*z* 106.0410 ([M-H-CO_2_-CH_3_-CO]^−^), enabling their identification as trans-ferulic acid and cis-ferulic acid, respectively.

In addition, 5 diferulic acid derivatives were identified in the bound phenolics based on their retention times and tandem mass spectrometry data in Table 1. Compound 30 (tR = 10.81 min) exhibited a deprotonated molecular ion [M-H]- at *m*/*z* 385.0933 in negative ionization mode. Its MS/MS spectrum revealed successive neutral losses, yielding fragment ions at [M-H-CO_2_]^−^, [M-H-CO_2_-CH_3_]^−^, [M-H-2CO_2_]^−^ and [M-H-2CO_2_-2CH_3_]^−^ ions at *m*/*z* 341.1035, 326.0797, 282.0897 and 267.0064. Thus, it was identified as 8-8′-Diferulic acid. Compound 35 (tR = 14.064 min) displayed a deprotonated molecular ion [M-H]^−^ at *m*/*z* 385.0934 in negative ionization mode. The MS/MS fragmentation pattern revealed sequential neutral losses, producing diagnostic ions at [M-H-CO_2_]^−^, [M-H-CO_2_-CH_3_]^−^, [M-H-CO_2_-2CH_3_]^−^, [M-H-2CO_2_]^−^ and [M-H-2CO_2_-2CH_3_]^−^ ions at *m*/*z* 341.1035, 326.0797, 311.0563, 282.0897 and 267.0064. Therefore, it was tentatively identified as 8-5′-Diferulic acid. The five diferulic acid derivatives were identified, indicating the structural diversity of bound phenolics in barley malt. The fragmentation patterns observed for compounds 30 and 35 suggest the presence of cross-linked ferulic acid dimers, which are typically associated with cell wall structures.

These results indicate that bound phenolics in barley malt are structurally diverse and largely composed of hydroxycinnamic acid derivatives, which may serve as key precursors for subsequent transformation during roasting. And these results provide a critical foundation for the subsequent multivariate metabolomic analysis and activity-based screening described below.

### 3.2. Nontargeted Metabolomics Analysis by UPLC-Q-Orbitrap HRMS/MS

Based on the chemical profiling results above, a non-targeted metabolomics approach was further employed to elucidate the dynamic changes of bound phenolics under different roasting conditions. UPLC-Q-Orbitrap HRMS/MS was used to analyze samples from raw, fried, and dark malt, and the acquired data were processed using Compound Discoverer 3.0, followed by normalization and multivariate statistical analysis using SIMCA-P 14.1. To comprehensively understand the changes in bound phenolic compounds at different roasting temperatures of barley malts, a non-targeted metabolomics analysis using UPLC-Q-Orbitrap HRMS/MS was employed to monitor metabolite variations in samples from three varieties of raw malt, fried malt and dark malt [29,30]. The raw data were processed using Compound Discoverer 3.0. Ion chromatography peak areas before and after processing, as well as across different processing methods, were integrated. The data were then uniformly processed using a normalization method and subsequently analyzed with SIMCA-P 14.1 multivariate statistical software.

**Figure 3:**
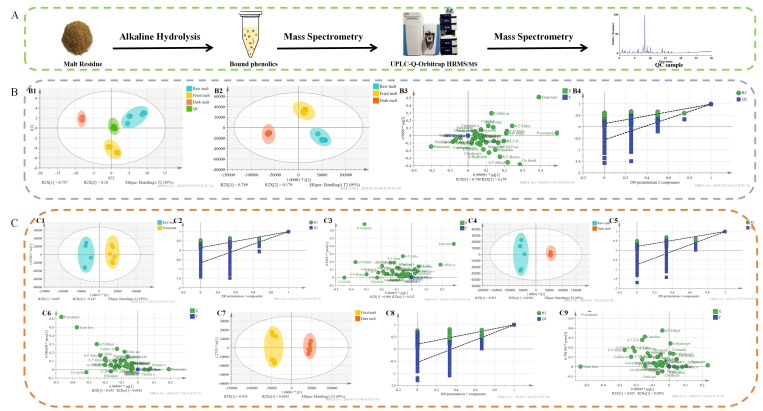
(**A**) The experimental design of metabolomics, (**B**) Multivariate statistical analyses of raw malt, fried malt and black malt. (**B1**) PCA score plots, (**B2**) OPLS-DA score plots, (**B3**) S-plots, (**B4**) 200× permutation test of OPLS-DA mode. (**C**) Screening of differential components for raw malt, fried malt and black malt. (**C1**–**C3**) OPLS-DA score plots of raw malt and fried malt, with S-plots and 200× permutation test; (**C4**–**C6**) OPLS-DA score plots of raw malt and black malt, with S-plots and 200× permutation test; (**C7**–**C9**) OPLS-DA score plots of fried malt and black malt, with S-plots and 200× permutation test.

In metabolomic investigations utilizing UPLC-MS platforms, quality control specimens play a pivotal role in verifying analytical reproducibility and data reliability. Throughout this investigation, the robustness of bound phenolic extracts was demonstrated through characteristic chromatographic profiles acquired in negative ionization mode (Figure 3A). Principal component analysis revealed clear segregation between QC specimens and experimental groups, while tight clustering of QC replicates indicated exceptional instrumental stability during prolonged operation (Figure 3B). Overall, these findings confirm that the established UPLC-MS methodology exhibits outstanding reproducibility, generating reliable analytical results throughout the experimental investigation.

Principal component analysis (PCA) was first performed to explore the overall metabolic differences among raw, fried, and dark malts. As shown in Figure 3B, clear separation among the three sample groups was observed, suggesting compositional differences in bound phenolics associated with different roasting levels. This observation is consistent with the chemical profiling results described in Section 3.1.

To facilitate visualization of group differences and the identification of discriminative metabolites, supervised OPLS-DA analyses were further performed. The score plots showed distinct clustering patterns among sample groups. However, permutation tests yielded negative Q^2^ values, indicating limited predictive ability of the supervised models. Therefore, the OPLS-DA results were interpreted primarily as exploratory tools for metabolite discrimination rather than as evidence of strong predictive performance (Figure 3C). Based on the combination of PCA analysis, OPLS-DA visualization, and subsequent chemical characterization, several metabolites with VIP values greater than 1.0 were identified as potential contributors to sample differentiation.

It should be noted that the supervised OPLS-DA models exhibited negative Q^2^ values in permutation tests, indicating limited predictive ability. Therefore, these models were used primarily for exploratory visualization and metabolite selection rather than for predictive purposes. Accordingly, the interpretation of compositional differences among sample groups relied mainly on PCA analysis and subsequent chemical characterization. Future studies involving larger sample sizes and additional validation strategies are warranted to further improve the robustness of multivariate analyses.

The PCA and OPLS-DA score plots clearly differentiated the various samples [31]. The findings indicate that the roasting phases represent pivotal manufacturing steps inducing chemical transformations. To identify characteristic markers differentiating malt types and potential process indicators, an S-plot analysis was conducted. Subsequent application of the variable importance in projection (VIP) method enabled selection of the most discriminative phenolics based on their separation power in the multivariate model. Seventeen key metabolites with VIP scores >1.0 were identified as significant classification markers (Table 2), which were subsequently classified into specific phenolic groups and structural subcategories based on their chemical characteristics. Notably, in comparative analyses between fried and raw malt, cis-ferulic acid exhibited the highest VIP score (2.88), followed by 6,3′-dihydroxy-4,4′-dimethoxy-5-methylaurone (2.40), *p*-coumaric acid (2.01), 8-5′-Benzofuran Diferulic acid (1.93) and trans-ferulic acid (1.80). Meantime, when comparing dark malts with raw malt, *p*-coumaric acid demonstrated superior VIP scores (3.03), with trans-ferulic acid (2.41), cis-ferulic acid (2.04) and 6,3′-Dihydroxy-4,4′-dimethoxy-5-methylaurone (1.62) ranking sequentially lower. Seventeen metabolites with VIP values > 1.0 were identified as key discriminatory compounds. Notably, *p*-coumaric acid and ferulic acid isomers ranked among the most influential variables, suggesting that these phenolics play a central role in differentiating malt samples under different roasting conditions.

**Figure 4:**
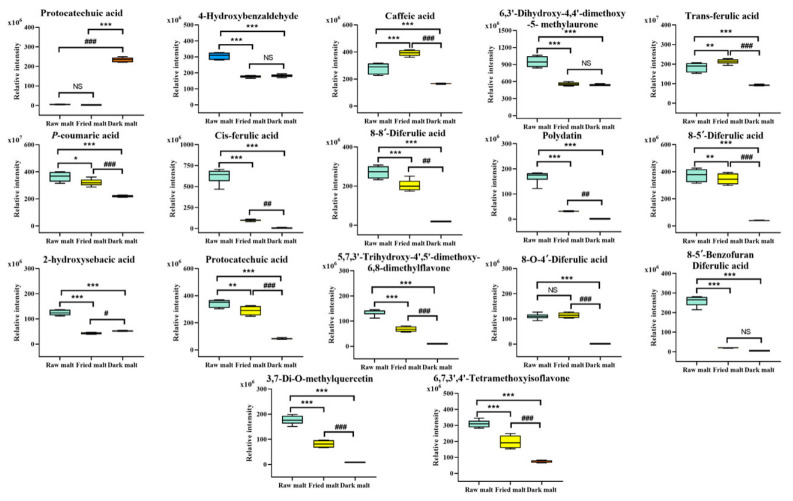
Differential markers screened by OPLS-DA in malt from the bound phenolics extracted by alkaline hydrolysis in three malt. (* *p* < 0.05, ** *p* < 0.01, *** *p* < 0.001 vs. Raw malt; ^#^ *p* < 0.05, ^##^ *p* < 0.01, ^###^ *p* < 0.001 vs. Fried malt; NS, not significant (*p* > 0.05)).

As shown in Figure 4, the peak areas of most bound phenolics decreased progressively with increasing roasting intensity. This trend suggests that changes in bound phenolics may be associated with thermal processing. The observed decrease in bound phenolics is consistent with previously reported phenomena involving the release, degradation, or transformation of phenolic compounds during roasting. The increase in protocatechuic acid suggests the possible occurrence of compound-specific transformation pathways during roasting. However, the formation routes of this compound were not directly monitored and therefore require further investigation. Several limitations of the present study should be acknowledged. Although raw, fried, and dark malt samples were obtained from the same manufacturer and from similar production batches to minimize variability, detailed information regarding roasting duration, moisture content, and germination conditions was not available. Consequently, the compositional differences observed among the samples cannot be attributed exclusively to roasting intensity, and the potential influence of other processing factors and batch-to-batch variability cannot be completely excluded. Therefore, the present findings should be interpreted with caution. Future studies using controlled malting and roasting systems are warranted to elucidate the specific effects of thermal treatment on the release, transformation, and antioxidant properties of bound phenolic compounds. Taken together, these metabolomic results not only validate the structural findings in Section 3.1 but also highlight roasting-induced chemical transformations, thereby providing mechanistic insight for the subsequent antioxidant activity screening.

### 3.3. Screening of Antioxidants by DPPH/AAPH-Incubating UPLC-DAD

Building on our previous findings demonstrating superior antioxidant capacity of bound phenolics in malt extracts via DPPH and ORAC assays, this study employs an optimized DPPH/AAPH-Incubating UPLC-DAD to systematically screen and quantify key antioxidant constituents within malt-bound phenolics [4,18,23].

**Figure 5:**
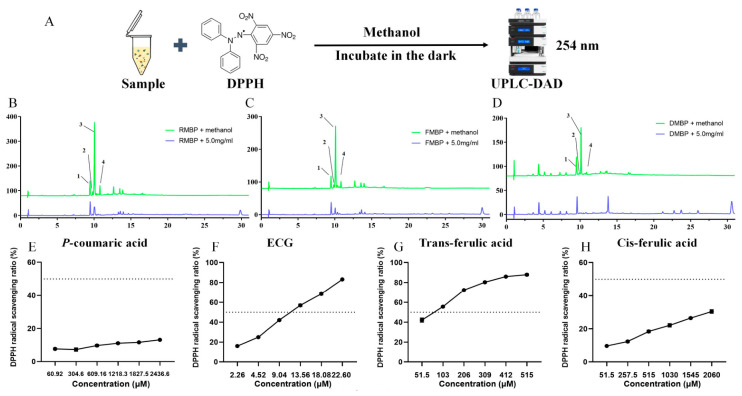
UPLC chromatogram of bound phenolic crude extract in DPPH-Incubating UPLC-DAD monitored at 254nm (**A**); RMPB and RMPB+DPPH (**B**), FMPB and FMPB+DPPH (**C**), DMPB and DMPB+DPPH (**D**). The DPPH radical-scavenging activity of *p*-coumaric acid (**E**), Epicatechin-gallate (**F**), trans-ferulic acid (**G**) and cis-ferulic acid (**H**). (RMPB: Raw malt bound phenolic. FMPB: Fried malt bound phenolic. DMPB: Dark malt bound phenolic. And the figure caption has been revised to include the following information: 1, *p*-coumaric acid; 2, epicatechin gallate; 3, trans-ferulic acid; 4, cis-ferulic acid. The dashed line indicates the 50% DPPH radical scavenging activity).

In this study, the antioxidants from bound phenolics extracted by alkaline hydrolysis in three malt were screened by the DPPH-Incubating UPLC-DAD assay. The DPPH-Incubating UPLC-DAD analysis enabled selective identification of antioxidant constituents through radical scavenging reactions. During this process, antioxidant compounds present in the extracts underwent oxidation upon interaction with DPPH radicals, resulting in either diminished or eliminated chromatographic peaks. In contrast, non-reactive components maintained stable peak intensities throughout the analysis [32,33]. This validated approach was subsequently applied to evaluate the bound phenolics isolated from three distinct barley malt varieties (Figure 5A). The DPPH-Incubating UPLC-DAD technique effectively discriminated potent radical scavengers from inactive constituents based on characteristic peak area variations.

Chromatographic analysis revealed that several peaks exhibited significant reductions after DPPH treatment, indicating strong radical scavenging capacity. Among these, *p*-coumaric acid, epicatechin gallate, and ferulic acid isomers were identified as compounds exhibiting strong radical-scavenging activity in Figure 5B–D. Meanwhile, we validated the DPPH radical-scavenging activity of *p*-coumaric acid, Epicatechin-gallate, trans-ferulic acid and cis-ferulic acid through in vitro experiments. The results showed that the IC50 for Epicatechin-gallate in scavenging DPPH radicals was 11.94 μM (Figure 5F), while the IC50 for trans-ferulic acid was 80.96 μM (Figure 5G). Interestingly, cis-ferulic acid as an isomer of trans-ferulic acid, exhibited a DPPH radical-scavenging IC50 greater than 2000 μM, indicating a weaker scavenging activity (Figure 5H). Additionally, *p*-coumaric acid showed even weaker DPPH radical-scavenging activity compared to cis-ferulic acid (Figure 5E). In vitro validation showed that epicatechin gallate and trans-ferulic acid exhibited strong DPPH radical scavenging activity, whereas cis-ferulic acid and *p*-coumaric acid showed weaker effects. Previous studies demonstrated that ferulic acid exhibited markedly stronger DPPH radical scavenging activity than isoferulic acid. Karamać et al. reported that isoferulic acid showed almost no antiradical activity at concentrations ranging from 10 to 100 nmol/assay, which was attributed to the different position of the hydroxyl group on the aromatic ring. The para-hydroxyl configuration in ferulic acid allows the formation of a larger number of resonance structures and facilitates hydrogen atom donation, thereby enhancing radical stabilization and DPPH scavenging activity [34]. This difference suggests that molecular structure, particularly the degree of conjugation and configuration, plays an important role in determining antioxidant capacity.

**Figure 6:**
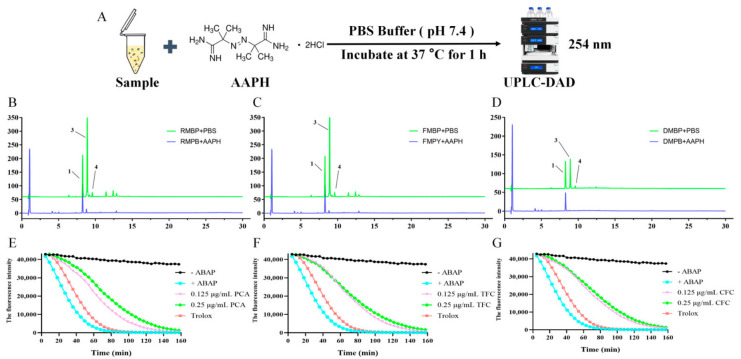
UPLC chromatogram of bound phenolic crude extract in AAPH-Incubating UPLC-DAD monitored at 254nm (**A**); RMPB and RMPB+AAPH (**B**), FMPB and FMPB+AAPH (**C**), DMPB and DMPB+AAPH (**D**). The ORAC assay of *p*-coumaric acid (**E**), trans-ferulic acid (**F**) and cis-ferulic acid (**G**). (The figure caption has been revised to include the following information: 1, *p*-coumaric acid; 3, trans-ferulic acid; 4, cis-ferulic acid.)

AAPH, a widely employed free radical generator, undergoes thermal decomposition to yield carbon-centered radicals. These reactive intermediates rapidly combine with molecular oxygen, generating peroxyl radicals that remain soluble in aqueous media. Such oxygen-derived radicals serve as representative species for studying oxidative degradation processes in biological systems [35]. The radical generation occurs under physiologically relevant conditions (pH 7.4). Antioxidant constituents are hypothesized to function through direct scavenging of ROO• radicals, leading to disruption of their conjugated electron systems [36]. Consequently, when these bioactive compounds interact with AAPH-derived peroxyl radicals, their chromatographic peak areas diminish significantly. In contrast, non-reactive molecules maintain stable signal intensities, serving as internal references [37]. Figure 6 displays the chromatograms of bound phenolics extracts monitored at 254 nm, with and without AAPH incubation. Chromatographic analysis revealed significant reductions in peak intensity for multiple constituents in the bound phenolics (Figure 6B,D). Specifically, twelve chromatographic peaks detected at 254 nm demonstrated marked decreases in area, suggesting these compounds actively scavenged AAPH-generated peroxyl radicals. We validated the antioxidant activity of *p*-coumaric acid, trans-ferulic acid and cis-ferulic acid through the ORAC assay in Figure 6E–G. The ORAC assay revealed a different activity pattern from that observed in the DPPH assay, with cis-ferulic acid exhibiting the highest ORAC value, followed by trans-ferulic acid and p-coumaric acid. This discrepancy suggests that antioxidant behavior is strongly influenced by the assay system and the underlying reaction mechanism. Through AAPH-Incubating UPLC-DAD and ORAC assays, we found that cis-ferulic acid, trans-ferulic acid and *p*-coumaric acid were identified as compounds exhibiting pronounced peroxyl radical-scavenging activity. Interestingly, trans-ferulic acid exhibited stronger radical-scavenging activity than cis-ferulic acid in the DPPH assay, whereas the opposite trend was observed in the ORAC assay. This discrepancy may be attributed to the distinct mechanisms underlying the two assays. The DPPH assay predominantly involves single-electron transfer reactions in an organic medium, while the ORAC assay evaluates hydrogen atom transfer reactions against peroxyl radicals under aqueous conditions [38,39,40]. Therefore, differences in molecular configuration may affect electron delocalization, radical stabilization, and interactions with peroxyl radicals differently. These findings highlight that antioxidant activity is highly dependent on both molecular structure and assay system, emphasizing the importance of employing multiple analytical approaches when evaluating the antioxidant properties of phenolic compounds.

The discrepancy between DPPH and ORAC results suggests that different phenolic compounds may exhibit distinct antioxidant mechanisms depending on the type of radicals involved. Although activity-guided screening and in vitro assays identified several compounds exhibiting strong radical-scavenging activity, the relative contribution of individual phenolics to the overall antioxidant capacity of the extracts was not quantitatively evaluated. It should be noted that the present study did not include absolute quantification or contribution analyses of individual phenolic compounds. Therefore, the antioxidant-active compounds identified through activity-guided screening should be regarded as potential contributors rather than definitive determinants of the overall antioxidant capacity. Future studies employing targeted quantification and contribution analyses are warranted to establish quantitative structure–activity relationships.

In addition to antioxidant activity, the biological effects of phenolic compounds are closely related to their bioavailability, which is strongly influenced by molecular structure and molecular weight. Generally, low-molecular-weight phenolic acids, such as *p*-coumaric acid and ferulic acid, exhibit relatively high bioaccessibility and can be absorbed in the small intestine [41,42]. In contrast, larger and more complex phenolic compounds often display limited absorption and may require enzymatic transformation by the gut microbiota before exerting biological effects [43]. Furthermore, structural characteristics, including hydroxyl substitution patterns, conjugated double bonds, and molecular configuration, influence not only radical-scavenging activity but also metabolic fate and bioavailability. Therefore, the functional significance of phenolic compounds should be evaluated by considering both their chemical reactivity and their potential biological accessibility. Future studies are warranted to investigate the bioavailability and in vivo antioxidant effects of these compounds. Overall, these findings indicate that key phenolic compounds identified in Section 3.1 and Section 3.2 are also the primary contributors to antioxidant activity, highlighting a close relationship between phenolic composition and functional properties.

## 4. Conclusions

In this study, the effects of different roasting levels on bound phenolics and their antioxidant activity in barley malts were systematically investigated using an integrated analytical approach. A total of 44 bound phenolic compounds were tentatively characterized, with phenolic acids such as p-coumaric acid and ferulic acid isomers representing the major components. Distinct compositional differences were observed among raw, fried, and dark malts, with raw malt exhibiting relatively higher levels of bound phenolics. The accumulation of protocatechuic acid suggests that differences in roasting levels may be associated with the release and transformation of phenolic compounds during thermal processing. Furthermore, antioxidant evaluation demonstrated that trans-ferulic acid, cis-ferulic acid, and *p*-coumaric acid exhibited different radical-scavenging activities. These differences highlight the influence of molecular structure on antioxidant behavior and emphasize the importance of employing complementary analytical methods to evaluate antioxidant properties.

Overall, this study provides new insights into the relationship between thermal processing, phenolic composition, and antioxidant activity in barley malts. These findings may contribute to the optimization of processing conditions for improving the functional quality of malt-based products.

Nevertheless, the mechanisms underlying the release and transformation of bound phenolics during thermal processing remain incompletely understood. The present findings further reinforce the need for mechanistic studies on phenolic release and transformation during thermal treatment. Future studies employing controlled roasting systems, kinetic analyses, and targeted characterization of intermediate products are warranted to elucidate the reaction pathways involved and to establish a clearer relationship between phenolic transformations, bioavailability, and biological functionality. Such investigations will contribute to a more comprehensive understanding of the health-promoting potential of thermally processed cereal products.

## Figures and Tables

**Table 1:** The identified Compounds extracted by alkaline hydrolysis in bound phenolics sample by UPLC-Q-Orbitrap HRMS/MS.

No.	MSI Level	Compound	RT	Expected	Measured	Adduction	Formula	Delta	MS2 ion
			(min)	(*m*/*z*)	(*m*/*z*)			(ppm)	(*m*/*z*)
1.	2	Citric acid	1.56	191.0191	192.0270	[M-H]^−^	C_6_H_8_O_7_	2.309	87.0072
2.	2	Protocatechuic acid	3.20	153.0183	154.0266	[M-H]^−^	C_7_H_6_O_4_	0.293	152.0102, 79.0176
3.	2	3,4-dihydroxyphenylacetic acid	3.35	167.0342	168.0423	[M-H]^−^	C_8_H_8_O_4_	1.765	122.0362, 123.0439
4.	2	2-Hydroxyisophthalic acid	3.64	181.0499	182.0215	[M-H]^−^	C_9_H_10_O_4_	1.683	121.0283, 119.0491, 108.0205, 93.0334
5.	1	Syringic acid	3.64	197.0450	198.0528	[M-H]^−^	C_9_H_10_O_5_	2.843	123.0441
6.	2	Isovanillin	4.39	151.0390	152.0473	[M-H]^−^	C_8_H_8_O_3_	0.128	108.0206, 93.0333
7.	2	Propyl gallate	4.39	211.0606	212.0684	[M-H]^−^	C_10_H_12_O_5_	2.132	136.0154, 108.0204, 59.0124
8.	2	4-Hydroxybenzoic acid	4.83	137.0232	138.0317	[M-H]^−^	C_7_H_6_O_3_	−0.953	93.0332
9.	2	Protocatechuic acid	4.94	153.0184	154.0266	[M-H]^−^	C_7_H_6_O_4_	0.424	124.0154, 79.0176
10.	2	Catechin	5.14	289.0717	290.0790	[M-H]^−^	C_15_H_14_O_6_	3.651	245.0814, 205.0502, 179.0345
11.	2	Homovanillic acid	5.70	181.0500	182.0579	[M-H]^−^	C_9_H_10_O_4_	2.733	137.0347, 122.0237
12.	2	4-Hydroxybenzaldehyde	6.23	121.0282	122.0367	[M-H]^−^	C_7_H_6_O_2_	−2.115	108.0204, 93.0333
13.	1	Caffeic acid	6.36	179.0342	180.0423	[M-H]^−^	C_9_H_8_O_4_	1.702	135.0444
14.	1	Vanillic acid	6.57	167.0343	168.0423	[M-H]^−^	C_8_H_8_O_4_	2.663	152.0107
15.	2	Phthalic acid	6.72	165.0186	166.0266	[M-H]^−^	C_8_H_6_O_4_	−1.415	NA
16.	1	Epicatechin	6.95	289.07214	290.0790	[M-H]^−^	C_15_H_14_O_6_	5.104	245.0814, 205.0502, 179.0345
17.	2	Vanillic aldehyde	7.09	151.0393	152.0473	[M-H]^−^	C_8_H_8_O_3_	1.850	136.0152, 108.0206
18.	2	Cerarvensin	7.17	401.0883	402.0950	[M-H]^−^	C_20_H_18_O_9_	3.943	218.0216
19.	2	6,3′-Dihydroxy-4,4′-dimethoxy-5- methylaurone	7.67	327.0877	328.0947	[M-H]^−^	C_18_H_16_O_6_	4.358	327.0923, 119.0491
20.	1	*P*-coumaric acid	8.21	163.0392	164.04734	[M-H]^−^	C_9_H_8_O_3_	1.100	119.0490, 107.0494, 93.0333
21.	2	Sigmoidin G	8.68	387.1090	388.1158	[M-H]^−^	C_20_H_20_O_8_	4.045	193.0500, 134.0363
22.	2	(-)—Epicatechin—3—(3″-O-methyI) gallate	8.70	455.0966	456.1056	[M-H]^−^	C_23_H_20_O_10_	−1.435	NA
23.	1	Epicatechin-gallate	8.99	441.0808	442.0900	[M-H]-	C_22_H_18_O_10_	−1.843	289.0719, 245.0818
24.	1	Chlorogenic acid	9.15	353.0877	354.0950	[M-H]^−^	C_16_H_18_O_9_	2.751	NA
25.	1	Trans-ferulic acid	9.21	195.0500	194.0579	[M-H]^−^	C_10_H_10_O_4_	2.563	134.0366, 106.0410
26.	2	Cinnamic acid	9.40	147.0443	148.0524	[M-H]^−^	C_9_H_8_O_2_	1.523	NA
27.	1	Cis-ferulic acid	10.10	193.0500	194.0579	[M-H]^−^	C_10_H_10_O_4_	2.563	134.0366, 106.0410
28.	2	Sinapic acid	10.51	223.0610	224.0685	[M-H]^−^	C_11_H_12_O_5_	4.125	193.0137, 149.0234
29.	2	8-8′-Diferulic acid	10.81	385.0933	386.1002	[M-H]^−^	C_20_H_18_O_8_	3.885	341.1035, 326.0797, 282.0897, 267.0664,
30.	2	Polydatin	11.79	389.1246	390.1314	[M-H]^−^	C_20_H_22_O_8_	3.639	267.0664
31.	2	2-Hydroxysebacic acid	12.11	217.1078	218.1154	[M-H]^−^	C_10_H_18_O_5_	3.454	NA
32.	2	5-5/8-8(cyclic)-TriFA	13.05	577.1360	578.1424	[M-H]^−^	C_30_H_26_O_12_	3.253	489.1561, 355.0810, 193.0501
33.	2	Azelaic acid	13.88	187.0960	188.1049	[M-H]^−^	C_9_H_16_O_4_	2.857	97.0649
34.	2	8-5′-Diferulic acid	14.06	385.0934	386.1002	[M-H]^−^	C_20_H_18_O_8_	4.275	326.0797, 311.0563, 282.0897, 267.0664
35.	2	8-8(cyclic)/5-5-TriFA	13.05	577.1360	578.1424	[M-H]^−^	C_30_H_26_O_12_	3.357	489.1561, 355.0810, 193.0501
36.	2	5-5′-Diferulic acid	15.96	385.0933	386.1002	[M-H]^−^	C_20_H_28_O_8_	3.885	341.1035
37.	2	8-O-4′-Diferulic acid	16.85	385.0934	386.1002	[M-H]^−^	C_20_H_18_O_8_	4.041	193.0501, 178.0263, 149.0599, 134.0363
38.	2	8-O-4/8-5(noncyclic)-TriFA	17.27	577.1358	578.1424	[M-H]^−^	C_30_H_26_O_12_	3.028	489.1561, 355.0810, 193.0501
39.	2	8-5′-Benzofuran Diferulic acid	17.39	385.0933	386.1002	[M-H]^−^	C_20_H_28_O_8_	3.963	326.0728, 311.0568, 267.0662, 239.0713
40.	2	8-8(cyclic)/8-O-4-TriFA	17.92	577.1359	578.1424	[M-H]^−^	C_30_H_26_O_12_	3.253	489.1561, 355.0810, 193.0501
41.	2	5,7,3′-Trihydroxy-4′,5′-dimethoxy-6,8-dimethylflavone	20.02	357.0985	358.1053	[M-H]^−^	C_19_H_18_O_7_	4.482	NA
42.	2	Apigenin	21.34	269.0457	270.0528	[M-H]^−^	C_15_H_10_O_5_	4.832	NA
43.	2	3,7-Di-O-methylquercetin	21.98	329.0672	330.0739	[M-H]^−^	C_17_H_14_O_7_	4.834	271.0239, 299.0202
44.	2	6,7,3′,4′-Tetramethoxyisoflavone	22.51	341.1033	342.1103	[M-H]^−^	C_19_H_18_O_6_	4.003	NA

**Table 2:** Processing Critical Metabolites (VIP > 1.00) to Variations in Metabolomics from Malt Roasting Processes.

No.	Compound	RT	Expected	Formula	Delta	MS2 ion	VIP		
		(min)	(*m*/*z*)		(ppm)	(*m*/*z*)	Raw Malt/ Fried Malt	Raw Malt/ Black Malt	Fried Malt/ Black Malt
1.	Protocatechuic acid	3.20	153.0183	C_7_H_6_O_4_	0.293	152.0102, 79.0176	VIP < 1.00	1.23426	1.51821
2.	4-Hydroxybenzaldehyde	4.83	137.0232	C_7_H_6_O_3_	−0.953	93.0332	1.41951	VIP < 1.00	VIP < 1.00
3.	Caffeic acid	6.36	179.0342	C_9_H_8_O_4_	1.702	135.0444	1.23168	VIP < 1.00	1.49046
4.	6,3′-Dihydroxy-4,4′-dimethoxy-5-methylaurone	7.67	327.0877	C_18_H_16_O_6_	4.358	327.0923, 119.0491	2.4043	1.62442	VIP < 1.00
5.	*P*-coumaric acid	8.21	163.0392	C_9_H_8_O_3_	1.100	119.0490, 107.0494, 93.0333	2.00997	3.03041	3.11083
6.	Trans-ferulic acid	9.20	195.0634	C_10_H_10_O_4_	0.844	149.0597, 145.0288, 117.0337, 89.0392	1.80334	2.40923	3.47297
7.	Cis-ferulic acid	10.10	195.0654	C_10_H_10_O_4_	2.563	149.0597, 145.0288, 117.0337, 89.0392	2.88354	2.03789	VIP < 1.00
8.	8-8′-Diferulic acid	10.81	385.0933	C_20_H_18_O_8_	3.885	341.1035, 326.0797, 282.0897, 267.0664,	VIP < 1.00	1.29322	1.34546
9.	Polydatin	11.79	389.1246	C_20_H_22_O_8_	3.639	267.0664	1.47163	1.05716	VIP < 1.00
10	2-hydroxysebacic acid	12.11	217.1078	C_10_H_18_O_5_	3.454	NA	1.1212	VIP < 1.00	VIP < 1.00
11.	8-5′-Diferulic acid	14.06	385.0934	C_20_H_18_O_8_	4.275	326.0797, 311.0563, 282.0897, 267.0664	VIP < 1.00	1.48374	1.737
12.	5-5′-Diferulic acid	15.96	385.0933	C_20_H_28_O_8_	3.885	341.1035	VIP < 1.00	1.30883	1.41295
13.	8-O-4′-Diferulic acid	16.85	385.0934	C_20_H_18_O_8_	4.041	193.0501, 178.0263, 149.0599, 134.0363	VIP < 1.00	VIP < 1.00	1.05964
14.	8-5′-Benzofuran Diferulic acid	17.39	385.0933	C_20_H_28_O_8_	3.963	326.0728, 311.0568, 267.0662, 239.0713	1.93104	1.30394	VIP < 1.00
15.	5,7,3′-Trihydroxy-4′,5′-dimethoxy-6,8-dimethylflavone	20.02	357.0985	C_19_H_18_O_7_	4.482	NA	1.02386	VIP < 1.00	VIP < 1.00
16.	3,7-Di-O-methylquercetin	21.98	329.0672	C_17_H_14_O_7_	4.834	271.0239, 299.0202	1.21243	1.06208	VIP < 1.00
17.	6,7,3′,4′-Tetramethoxyisoflavone	22.51	341.1033	C_19_H_18_O_6_	4.003	NA	1.25957	1.2463	1.0486

NA: not assessed.

## Data Availability

The original contributions presented in this study are included in the article. Further inquiries can be directed to the corresponding authors.

## Abbreviations

The following abbreviations are used in this manuscript:

AAPH	2,2′-Azobis(2-amidinopropane) dihydrochloride
CD	Compound Discover
DPPH	2,2-diphenyl-1-picrylhydrazyl
OPLS-DA	Orthogonal Partial Least Squares Discriminant Analysis
PCA	Principal Component Analysis
